# Zerumbone-Loaded Nanostructured Lipid Carrier Induces Apoptosis of Canine Mammary Adenocarcinoma Cells

**DOI:** 10.1155/2018/8691569

**Published:** 2018-10-15

**Authors:** Jia Ning Foong, Gayathri Thevi Selvarajah, Abdullah Rasedee, Heshu Sulaiman Rahman, Chee Wun How, Chaw Yee Beh, Guan Young Teo, Chi Ling Ku

**Affiliations:** ^1^Department of Veterinary Clinical Studies, Faculty of Veterinary Medicine, Universiti Putra Malaysia, 43400 UPM Serdang, Malaysia; ^2^Laboratory of Vaccine and Immunotherapeutics, Institute of Bioscience, Universiti Putra Malaysia, 43400 UPM Serdang, Malaysia; ^3^Department of Veterinary Laboratory Diagnosis, Faculty of Veterinary Medicine, Universiti Putra Malaysia, 43400 UPM Serdang, Malaysia; ^4^College of Veterinary Medicine, University of Sulaimani, Sulaimani City, Iraq; ^5^Faculty of Pharmacy, Mahsa University Malaysia, 42610 Jenjarum, Selangor, Malaysia

## Abstract

Canine mammary gland tumor (CMT) is the most common tumor in intact female dog. Zerumbone (ZER) has promising anticancer properties, but plagued with poor water solubility, poor absorption, bioavailability, and delivery to target tissues. To solubilize, ZER was loaded into nanostructured lipid carrier (NLC) to produce ZER-loaded NLC (ZER-NLC). The objectives of this study were to determine the antiproliferative effect and the mode of cell death induced by ZER-NLC and ZER on a canine mammary gland tumor (CMT) adenocarcinoma primary cell line. There was no significant difference (*p*>0.05) between ZER-NLC and ZER treatments in the inhibition of CMT cell proliferation; thus, the loading of ZER into NLC did not compromise the cytotoxic effect of ZER. Microscopically, ZER-NLC- and ZER-treated CMT cells showed apoptotic cell morphology. ZER-NLC and ZER treatments significantly downregulated the antiapoptotic Bcl-2 and upregulated the proapoptotic Bax gene expressions in CMT cells. Both ZER-NLC and ZER-treated CMT cells showed significant (*p*<0.0001) increases in caspase-8, -9, and -3/7 protein activities. In conclusion, ZER-NLC induced CMT cell death via regulation of Bcl-2 and Bax gene expressions and caspase activations, indicating the involvement of both the intrinsic and extrinsic pathways of apoptosis. This study provided evidences for the potential of ZER-NLC as an anticanine mammary gland adenocarcinoma chemotherapy.

## 1. Introduction

Canine mammary gland tumor (CMT) is the most common tumor in intact female dogs [1–3]. Approximately 50% of CMT cases diagnosed are malignant and most often require surgical resection [4, 5]. Current chemotherapeutic drugs for the treatment of CMT like doxorubicin or cisplatin are nonselective, toxic, and with severe adverse effects. Many phytochemicals including flavonoids, cannabinoids, polyenes, carotenoids, and alkaloids have also been shown to have anti-breast cancer properties [6]. However, the effect of these phytochemicals on animal mammary gland tumors is not known.

The edible wild ginger* Zingiber zerumbet *(L.) Smith contains several phytochemicals with healing properties. This herbal plant is traditionally used to treat gastrointestinal disorders, intestinal worms, toothaches, fever, nausea, arthritis, and sprains [7, 8]. Zerumbone (ZER) is a major lipophilic compound isolated from the essential volatile oil of* Zingiber zerumbet *(L.) Smith rhizomes [9–12]. ZER possesses antitumor, anti-inflammatory, antioxidant, antimicrobial, antinociceptive, hepatoprotective, and immunomodulatory activities [10, 13, 14]. In spite of these effects, the therapeutic application of ZER is plagued by poor water solubility with subsequent poor absorption, bioavailability, and delivery to target tissues and organs [10, 15].

Nanoparticles have great potential to be developed as drug carriers, especially for poor water-soluble compounds. The nanostructured lipid carriers (NLC), for example, have the capacity to be loaded with lipophilic drugs for enhanced delivery, controlled-release, increased drug tolerability, and flexibility of administration [16–21]. NLC is composed of solid lipid matrix incorporated into liquid lipids, producing crystalline imperfections that allow for greater drug entrapment [18, 21]. The NLC is a nanometer-sized drug-delivery system with superior particle surface-to-volume ratio, high loading efficiency, and drug bioavailability [19] and allows for the solubilization of lipophilic substances like ZER for parenteral administrations.

ZER-loaded NLC (ZER-NLC) has apoptogenic effect on acute human lymphoblastic leukemia (Jurkat) cell line [10], murine myelocytic leukemia cell line (WEHI-3B) [22], human mammary adenocarcinoma (MDA-MB-231) cell line [23], and murine breast cancer (4T1) cell line [24]. The anticancer effect of ZER-NLC on canine mammary gland tumor (CMT) is not known. Thus, the objectives of this study were to determine the antiproliferative effect of ZER-NLC and ZER on a canine mammary gland tumor adenocarcinoma primary cell line and the mode of tumor cell death induced by ZER-NLC and ZER.

## 2. Materials and Methods

### 2.1. Cell Line and Culture Conditions

**Figure 1:**
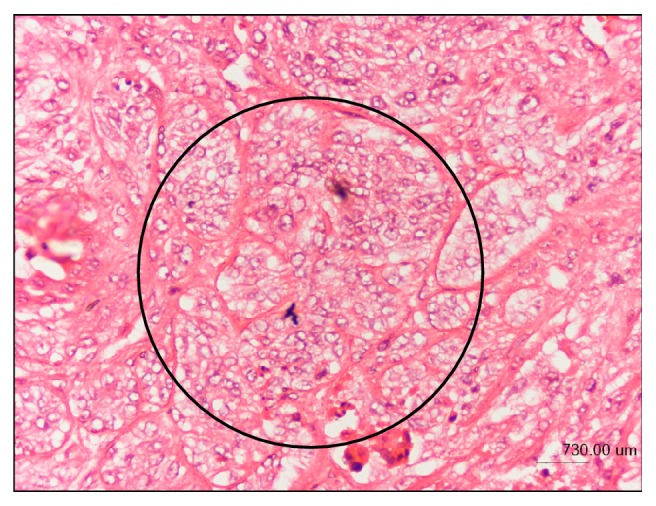
Canine mammary gland tumor tissue biopsy showing poorly differentiated epithelial cells (black circle) and marked anisocytosis and anisokaryosis (H&E staining).

**Figure 2:**
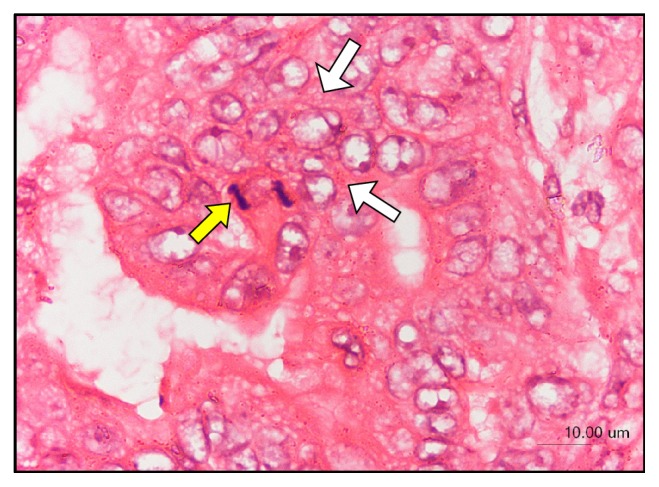
Canine mammary gland tumor tissue biopsy showing high cellularity with evidence of infiltrative growth (white arrows), mitosis (yellow arrow), marked anisocytosis and anisokaryosis, prominent nucleoli, and clear cytoplasmic vacuoles (H&E staining).

A canine mammary gland tumor (CMT) primary cell line was obtained from a surgically excised stage 4 CMT of a 13-year-old spayed female dog of Malaysian local breed presented to the University Veterinary Hospital, Universiti Putra Malaysia. The mandibular lymph nodes and popliteal lymph nodes of the dog were mildly enlarged, and ultrasonography revealed liver mineralization and spleen mass, which are evidences of metastasis. Tumor biopsies were obtained for histology assessment using hematoxylin and eosin (H&E) staining. Tumor paraffin section of the CMT showed poorly differentiated epithelial cells (Figure 1), marked anisocytosis, anisokaryosis, evident nucleoli, clear cytoplasmic vacuoles, and high cellularity with evidence of mitosis and infiltrative growth (Figure 2), indicative of advanced tumor stage.

Cells were dissociated into suspension from excised mammary gland tumor tissue samples via enzymatic digestion in prewarmed 2% Trypsin-EDTA solution (Sigma-Aldrich®, USA) for 4 h. The cell suspension was then centrifuged at 4°C, 200 ×* g* for 10 min, and the supernatant was discarded. The cell pellet was resuspended with fresh Roswell Park Memorial Institute 1640 medium (RPMI) (Gibco™, USA) growth medium containing 10% fetal bovine serum and incubated at 37°C under 5% CO_2_ in T-25 cm^2^ flasks (TPP®, Sigma-Aldrich®, USA). The removal of fibroblastic stromal cells from the tumor cell mixture was by the selective attachment method [25]. This was done by seeding the cell suspension in a T-25 cm^2^ flask for 1 h. Unattached cells were harvested and placed in a fresh T-25 cm^2^ flask. This process was repeated every 24 h until all visible fibroblasts were removed. The presence of visible fibroblast was determined by examination under a microscope at 400× magnification and confirmed with reverse transcriptase polymerase chain reaction (RT-PCR). Finally, the dissociated cells were maintained in fresh Roswell Park Memorial Institute 1640 medium (RPMI) (Gibco™, USA) supplemented with 10% fetal bovine serum (HyClone™, USA), 100 units/mL penicillin, and 100 *μ*g/mL streptomycin (Gibco™, USA) at 37°C under 5% CO_2_ in humidified atmosphere.

### 2.2. Characterization of Canine Mammary Gland Tumor Primary Cell Line

The cells from the tumor were characterized by reverse transcription polymerase chain reaction (RT-PCR) gene expression via mammary gland tumor molecular markers identification [26]. Purified total RNA was obtained from the CMT cells to generate first-strand cDNA via Reverse Transcriptase that served as template for subsequent polymerase chain reaction (PCR). MasterPure™ RNA Purification Kit (Epicentre® Biotechnologies, USA) was used for total RNA isolation. Reverse transcription of the RNA was carried out using Maxime RT Premix (iNtRON Biotechnology, Korea). The synthesized cDNA was then used as template for PCR with i-PCR Master Mix (i-DNA, Singapore). Amplified DNA was detected by ethidium bromide staining in 2% agarose gel. The size of the amplified DNA was determined using GeneRuler™ 100bp DNA Ladder (Fermentas Life Sciences, USA). A normal non-cancerous cell line, the 3T3 Murine fibroblast (ATCC, USA), was used for comparison.

Ten genes were analyzed, namely, cytokeratin-8 (CK-8), hypoxanthine ribosyltransferase (HPRT), estrogen receptor (ER), progesterone receptor (PGR), vascular endothelial growth factor (VEGF), human epidermal growth factor receptor-2 (HER-2), hypoxia-inducing factor-1*α* (HIF-1*α*), B-cell lymphoma-2 (Bcl-2), matrix metalloproteinases-2 (MMP-2), and erythropoietin receptor (EPOR). Hypoxanthine ribosyltransferase (HPRT) gene was used as reference. Primer sequences and their respective annealing temperature employed in the CMT cell characterization are shown in Table 1.

### 2.3. Zerumbone Extraction

**Figure 3:**
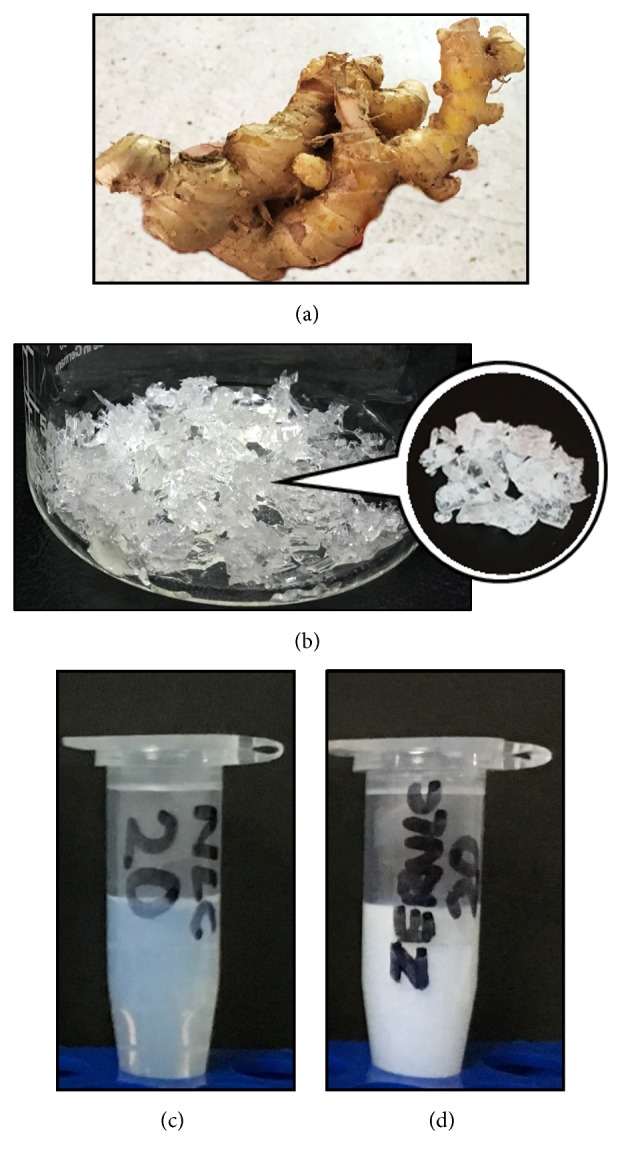
(a) Fresh* Zingiber zerumbet *(L.) Smith rhizomes, (b) pure colorless zerumbone (ZER) crystals, (c) nonloaded nanostructured lipid carrier (NLC) suspension, and (d) zerumbone-loaded nanostructured lipid carrier (ZER-NLC) suspension.

Fresh* Zingiber zerumbet *(L.) Smith ginger rhizomes were purchased from Kiza Herbs Farm, Pahang, Malaysia (Figure 3(a)). Crude ZER crystals were extracted from the essential oil of* Zingiber zerumbet *(L.) Smith rhizomes by hydrodistillation [10]. Recrystallization was performed thrice with absolute n-hexane (Sigma-Aldrich®, USA) to obtain pure colorless ZER crystals (Figure 3(b)).

### 2.4. Determination of ZER Purity via HPLC

The purity of ZER was determined using the calibrated and validated high performance liquid chromatography (HPLC) system (Waters, USA) [27]. Chromatographic separation was with the use of a reverse-phase Eclipse Plus C18 column (Agilent, USA) (5_m, 150 mm × 4.6 mm). The stationary phase was equilibrated with an isocratic mobile phase composed of methanol and formic acid (0.05%, v/v) (1:3). 1.0 *μ*L of 2.3 mg/mL ZER crystals was added to absolute methanol and injected at 1 mL/min flow rate. The sample was estimated under UV at 280 nm wavelength and the results were analyzed using the Empower Pro chromatography software (Waters, USA). The purity (%) of ZER was determined by calculating the area under the curve.

### 2.5. Preparation of Zerumbone-Loaded Nanostructured Lipid Carriers

NLC and ZER-NLC were prepared by high-pressure homogenization [10] (Figures 3(c) and 3(d)). The lipid phase, composed of hydrogenated palm oil (4%, w/v) (Softisan 154, MPOB, Malaysia), olive oil (1%, v/v) (Basso Fedele, Italy), and lecithin (1.73%, w/v) (Lipoid S100, GmbH & CO. KG, Germany), was melted by heating to 70°C before adding 500 mg ZER crystals. Sorbitol (4.75%, w/v), thimerosal (0.005%, w/v) (Sigma-Aldrich®, USA), and polysorbate 80 (1%, v/v) (Thermo Fisher Scientific®, USA) dissolved in double distilled-water to total volume of 100 mL were heated to the same temperature as the lipid matrices. Zerumbone (0.5%, w/v) in lipid melt was dispersed in the aqueous surfactant solution by high-speed stirring in a digital homogenizer (IKA® T25 Ultra Turrax®, Germany) at 13,000 rpm for 10 min, to obtain a hot pre-emulsion. The hot pre-emulsion was then homogenized in a high-pressure homogenizer (EmulsiFlex™, Avestin Inc, Ottawa, Canada) at 1,000 bars for 20 cycles at 60°C to obtain a hot oil-in-water nanoemulsion. The nanoemulsion was transferred to glass vials, sealed, and allowed to undergo lipid phase crystallization at room temperature (25°C) for the formation of ZER-NLC. NLC was similarly prepared but without addition of ZER.

### 2.6. Characterization of Zerumbone-Loaded Nanostructured Lipid Carriers

To determine long-term stability of ZER-NLC, three tubes of 5 mL ZER-NLC each were stored separately in the dark at 4°C, room temperature (24-26°C), and 40°C, respectively. Every 30 days for 9 months, 0.1 mL of each ZER-NLC suspension was diluted with 0.9 mL double-distilled water (1:10 dilution) and the z-average, polydispersity index (PDI), and zeta potential (ZP) were determined (Zetasizer Nano® ZS, Malvern™ Instrument Ltd, UK) [10, 28].

### 2.7. MTT Cell Viability Assay

The antiproliferative effect of NLC, ZER, and ZER-NLC on CMT cells (passage 10) was quantified by MTT (3-(4,5-dimethylthiazol-2-yl)-2,5-diphenyltetrazolium bromide) assay [10]. Cells grown to near confluence in T-75 cm^2^ flasks (TPP®, Sigma-Aldrich®, USA) were trypsinized and counted. 100 *μ*L of 0.2 × 10^4^ CMT cells in growth medium was seeded into each well of eight 96-well flat-base plate (Falcon®, Becton Dickinson Labware, USA) and the plate incubated overnight. Pure ZER stock was dissolved in DMSO (Sigma-Aldrich®, USA) and NLC and ZER-NLC working solutions in RPMI-1640 complete growth medium (Invitrogen Corp., Auckland, New Zealand).

Two plates were assigned to each incubation time of 0, 24, 48, and 72 h. The cells were treated with either 6.5, 12.5, 25, 50, or 100 *μ*M of doxorubicin (positive control), ZER, NLC, or ZER-NLC [23]. Negative control wells received 100 *μ*L RPMI-1640 growth medium only. DMSO served as vehicle control. The plates were then incubated accordingly for 24, 48, or 72 h before the cells subjected to MTT assay. Briefly, 20 *μ*L MTT stock solution (5 mg/mL, Sigma-Aldrich®, USA) in PBS was added to each well. The plates were then incubated in the dark for 3 h to allow viable cells to convert MTT to a water-insoluble purple formazan dye. The medium was aspirated and the remaining purple formazan dissolved in 100 *μ*L DMSO. The plates were then incubated for another 30 min and the absorbance was determined spectrophotometrically using an ELISA universal plate reader (Bio-Tek® Instruments Inc., Winooski, VT, USA) at 570 nm after background correction at 630 nm. The results were expressed as the percentage of cell proliferation with respect to the negative control. The half maximal growth inhibitory concentration (GI_50_), concentration that reduces the growth of treated cells by 50 % (i.e., x intercept where y = 50); total growth inhibition concentration (TGI), concentration that inhibits cell growth completely (i.e., x intercept where y = 0); and median lethal concentration (LC_50_), concentration that kills 50 % of the treated cells (i.e., x intercept where y = -50) were determined from the concentration-response curves [29].

### 2.8. Fluorescence Microscopy with Acridine Orange and Propidium Iodide Staining

Cells grown to near confluence in T-75 cm^2^ flasks were trypsinized and counted. 1 mL of suspension containing 6.0 × 10^4^ CMT cells was then seeded into each well of 6-well flat-based plates (Falcon®, Becton Dickinson Labware, USA) and the plates were incubated overnight. Then, the cells were treated with ZER and ZER-NLC at their respective 72-h LC_50_ concentrations before reincubation for 24, 48, or 72 h (Table 2). Since NLC did not record any 72-h GI_50_, TGI, or LC_50_ value, the concentrations of NLC used were that of ZER-NLC 72-h LC_50_ values. Negative control wells were treated with medium only. After 24 h, the cells were trypsinized, harvested, and centrifuged (Centrifuge 5810R, Eppendorf™, Germany) at 1000 rpm and 4°C for 10 min. The supernatant was discarded, and the cells were washed twice with PBS. 10 *μ*L of fluorescence dyes containing acridine orange (100 *μ*g/mL) (Invitrogen™, USA) and propidium iodide (100 *μ*g/mL) (Sigma-Aldrich®, USA) was added to each cell pellet and a drop of freshly stained cell suspension on a glass slide viewed under UV-fluorescence microscope (Zeiss™ Axio Vert. A1, USA). The procedure was repeated for cells incubated for 48 and 72 h. For each treatment, the percentage of viable, early apoptotic, late apoptotic, and necrotic cells to total number of cells was determined on at least 200 cells [30].

### 2.9. RNA Isolation

Cells grown to near confluence in T-75 cm^2^ flasks were trypsinized and counted. 5 mL suspension containing 1.6 × 10^5^ CMT cells was seeded into each T-25 cm^2^ flask (TPP®, Sigma-Aldrich®, USA) and the flask incubated overnight at 37°C under 5% CO_2_ humidified atmosphere. The cells were then treated with NLC at LC_50_ concentrations (72-h ZER-NLC LC_50_ equivalent concentration), ZER, or ZER-NLC, or with medium (negative control) (Table 2). The flasks were incubated for 3, 6, 9, 12, or 15 h and cells harvested for RNA isolation (Qiagen®, RNeasy® Plus Mini kit, Germany). The medium in the flask was removed and the cellsvwere washed twice with PBS. 350 *μ*L Buffer RLT plus containing *β*-mercaptorethanol (Bio-Rad Laboratories, Inc., USA) was added to each flask and cells were collected, using a cell scraper (TPP®, Sigma-Aldrich®, USA), into microcentrifuge tubes. The cell homogenates were transferred into a gDNA Eliminator Spin Column (Qiagen®, RNeasy® Plus Mini kit, Hilden, Germany) and centrifuged at 10,000 rpm for 30 s. One volume of 350 *μ*L 70% ethanol was added to the flow-through before transferring to RNeasy Spin Column and centrifugation at 10,000 rpm for 15 s and the flow-through discarded. 350 *μ*L Buffer RW1 was added to the spin column and then centrifuged at 10,000 rpm for 15 s and the flow-through discarded. 500 *μ*L Buffer RPE was added to the pellet, mixed, and centrifuged at 10,000 rpm for 2 min. The collection tube was then replaced with a new collection tube, centrifuged at full speed for 1 min. The flow-through and collection tube were discarded. The spin column was then placed in a new microcentrifuge tube, 30 *μ*L of double-distilled water added, and the column centrifuged at 10,000 rpm for 1 min. The spin column was discarded, and the isolated RNA was stored under -80°C until used. The RNA quality determination and quantification were done using a nanodrop machine (Infinite® 200 Pro, Tecan, Switzerland).

### 2.10. cDNA Synthesis

The cDNA synthesis from corresponding RNA was done using the QuantiNova™ Reverse Transcription Kit (Qiagen®, Germany). Freshly filtered double-distilled water, RNA at 1000 ng/reaction, Reverse Transcription Reaction Mix, and Reverse Transcription Enzyme were added to a 200 *μ*L PCR tube (Axygen™, Fisher Scientific®, USA). The tubes were then placed in a thermocycler (MyCycler™ Thermal Cycler System, Bio-Rad Laboratories Inc., USA) that was run for one cycle of 5 min at 25°C, 30 min at 42°C, and 5 min at 85°C. Finally, the samples were held at 16°C until collection. The cDNA was stored at -20°C.

### 2.11. Quantitative Real-Time PCR Assay

Two-step quantitative real-time PCR (qRT-PCR) was employed using QuantiNova™ SYBR® Green PCR Kit (Qiagen®, Germany) using primers for Bax, Bcl-2 [31], RPS-19, and GAPDH [32] (Table 3). The real-time thermocycler (CFX96™ Real-time Cycler, Bio-Rad Laboratories Inc., USA) was programmed at the primer-specific annealing temperatures as stipulated in the protocol of the kit. Internal standards at 2-fold dilutions were included for each gene to determine efficiency rate (E), coefficient of determination (R^2^), and slope rate of the PCR reaction. Melting curve analysis was included. The qRT-PCR analyses were performed in triplicate. The ZER-, NLC-, and ZER-NLC-treated CMT cell gene expressions were analyzed in CFX Manager™ software (Bio-Rad Laboratories Inc., USA) against the reference RPS-19 and GAPDH genes and compared to the negative control.

### 2.12. Caspase Luminescent Assay

Cells grown to near confluence in T-75 cm^2^ flasks were trypsinized and counted. 100 *μ*L suspension containing 0.2 × 10^4^ CMT cells was seeded into each well of 96-well microculture plates (BRAND®, GmbH & CO. KG, Germany). The plates were incubated overnight at 37°C under 5% CO_2_ humidified atmosphere, treated with 72-h LC_50_ concentrations of ZER-NLC, ZER, or 72-h ZER-NLC LC_50_ equivalent concentration of NLC, and incubated for 3, 6, 9, 12, 24, 48, or 72 h at 37°C under 5% CO_2_ humidified atmosphere. Negative control wells were treated with medium. 100 *μ*L of caspase-glo® 3/7, caspase-glo® 8, and caspase-glo® 9 reagents (Promega Co., USA) was added to the designated wells. The contents of wells were gently mixed at 300 rpm (Rotamax 120, Heidolph, GmbH & CO. KG, Germany) for 30 s and incubated at room temperature for 1 h. The luminescence was determined using a luminometer (Infinite® 200 Pro, Tecan Trading AG, Switzerland). The results were expressed as ratios of activated caspase protein to the negative control.

### 2.13. Statistical Analysis

The results were expressed as mean ± standard deviation (SD). Analysis of variance (ANOVA) and* Post hoc* Tukey test were performed using the SPSS version 20.0 software (Chicago, IL, USA) for all experiments performed. Probability value of* p* < 0.05 was used to determine significance.

## 3. Results

### 3.1. Molecular Markers of Canine Mammary Gland Tumor Cells

**Figure 4:**
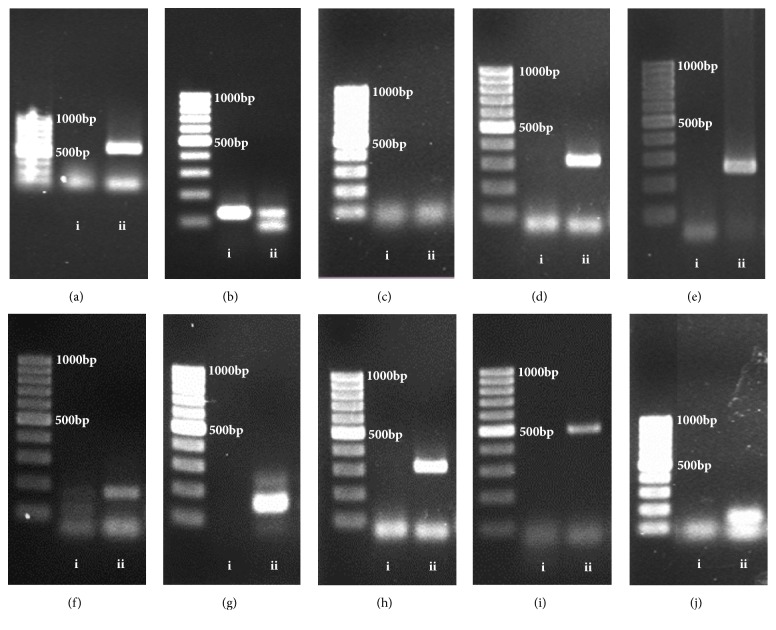
Gene expression in canine mammary gland tumor (CMT) adenocarcinoma cells. (a) Cytokeratin-8 (CK-8), (b) hypoxanthine ribosyltransferase (HPRT), (c) estrogen receptor (ER), (d) progesterone receptor (PGR), (e) vascular endothelial growth factor (VEGF), (f) human epidermal growth factor receptor-2 (HER-2), (g) hypoxia-inducing factor-1*α* (HIF-1*α*), (h) B-cell lymphoma-2 (Bcl-2), (i) matrix metalloproteinases-2 (MMP-2), (j) erythropoietin receptor (EPOR) (agarose gel, ethidium bromide staining). i = 3T3 murine fibroblasts; ii = CMT cells; bp = base pair.

The CMT cells were positive for CK-8, HPRT, PGR, VEGF, HER-2, HIF-1*α*, Bcl-2, MMP-2, and EPOR and negative for ER gene (Table 4). Gene expressions for CMT cells and 3T3 murine fibroblasts in agarose gel were shown in Figure 4. The CMT cells were identified as canine mammary gland tumor adenocarcinoma cells.

### 3.2. Purity of ZER

**Figure 5:**
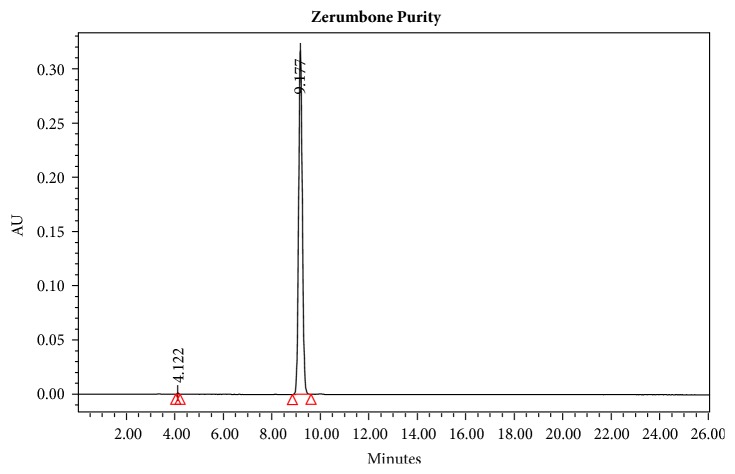
HPLC analysis of zerumbone (ZER) crystals showing a major peak at 9.177 min retention time.

The purity of ZER determined by the HPLC system was 99.82% showing a major peak with retention time of 9.177 min (Figure 5).

### 3.3. Characteristics of Zerumbone-Loaded Nanostructured Lipid Carriers

**Figure 6:**
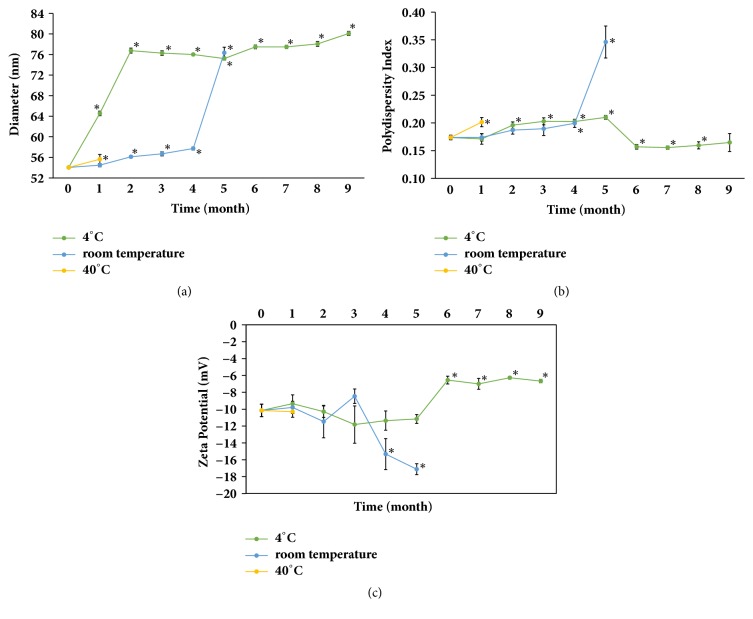
Long-term stability of ZER-NLC. (a) Particle size, (b) polydispersity index, and (c) zeta potential of zerumbone-loaded nanostructured lipid carrier (ZER-NLC) stored at various temperatures. For each temperature, means with asterisk (*∗*) are significantly (*p*<0.05) different from initial (0 month) mean.

The ZER-NLC formulation was a milky-white and semitransparent aqueous suspension (Figure 3(d)). The particle sizes of freshly prepared ZER-NLC were 54.04 ± 0.19 nm. ZER-NLC stored at 4°C increased in size significantly (*p*<0.0001) to 64.56 ± 0.44 nm and to 76.76 ± 0.49 nm after one and two months, respectively (Figure 6). Thereon, the particle size remained relatively constant reaching an average maximum of 80.09 ± 0.41 nm by the 9th month. The suspension remained semitransparent even by the 10th month of storage at 4°C. ZER-NLC samples stored at room temperature only began to show significant (*p*<0.0001) increases in size on the 5th month. However, by the 6th month of storage at room temperature, the suspension solidified.

The PDI of freshly produced ZER-NLC was 0.17 ± 0.004. Unlike particle size, there is no significant difference in PDI between samples stored at 4°C and room temperature during the first four months of storage. However, by the 5th month, the PDI of samples stored at room temperature was significantly (*p*<0.0001) higher than those stored at 4°C. For the first two months of storage the PDI of ZER-NLC at room temperature was fairly constant, between 0.17 ± 0.004 and 0.19 ± 0.007 before escalating to 0.35 ± 0.029 on the 5th month and solidifying by the subsequent month. The ZER-NLC at 4°C was relatively stable with PDI values ranging from 0.16 ± 0.003 to 0.21 ± 0.003.

The zeta potential (ZP) of freshly prepared ZER-NLC was -10.15 ± 0.75 mV. There was no significant difference in ZP between ZER-NLC samples stored at room temperature, 4°C, and 40°C for the first month of storage. The ZP of the 4°C ZER-NLC sample increased suddenly after the 5th month of storage to constant range of -7.00 ± 0.64 to -6.28 ± 0.15mV. ZER-NLC samples stored at 4°C remained as stable semitransparent suspension for at least 9 months of storage.

After one month of storage at 40°C, the ZER-NLC sample showed particle size of 55.62 ± 0.94, PDI of 0.20 ± 0.008, and zeta potential of -10.30 ± 0.66. After that, the 40°C ZER-NLC sample solidified into a whitish form; thus, no further analysis was made on the sample.

### 3.4. Cytotoxic Effects

**Figure 7:**
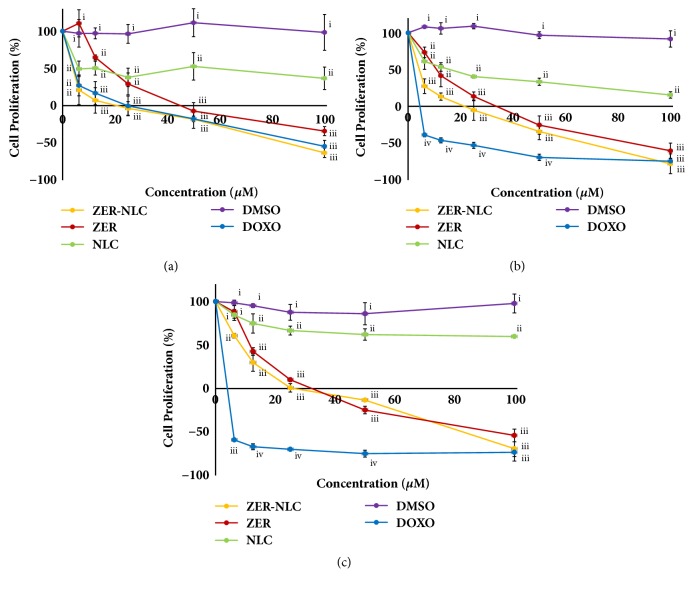
Cytotoxicity of ZER-NLC, ZER, NLC, DMSO, and DOXO treatments on canine mammary gland tumor (CMT) adenocarcinoma cells at (a) 24, (b) 48, and (c) 72 h. For each concentration, means with different roman numerals were significantly different (*p*<0.05) from each other. DMSO: dimethyl sulfoxide; DOXO: doxorubicin; NLC: nanostructured lipid carrier; ZER: zerumbone; ZER-NLC: zerumbone-loaded nanostructured lipid carrier.

The effects of ZER-NLC, ZER, NLC, and doxorubicin treatments on the viability and proliferation of CMT cells were concentration-dependent (Figure 7). Generally, there was no significant difference (*p*=0.606) in proliferation between cells treated with ZER-NLC and ZER for 72 h. The CMT cells treated with NLC lost some viability at 24 h, suggesting NLC is slightly cytotoxic to these cells. Doxorubicin was most cytotoxic among test compounds causing rapid and early loss of cell viability reaching low values at even the lowest concentrations of the drug.

**Figure 8:**
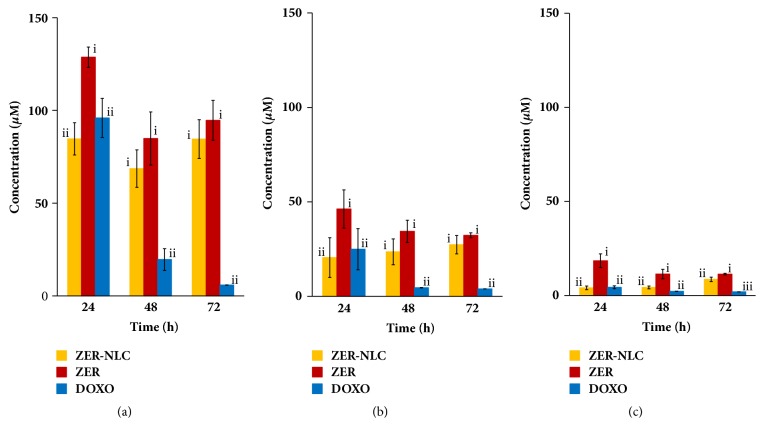
Toxicity of ZER-NLC, ZER, and doxorubicin on canine mammary gland tumor (CMT) adenocarcinoma cells. (a) Lethal concentration (LC_50_), (b) total growth inhibition concentration (TGI), and (c) growth inhibition concentration 50 (GI_50_) of ZER-NLC, ZER, and DOXO at 24, 48, and 72 h of incubation. For each incubation period, means with different roman numerals were significantly different (*p*<0.05) from each other. DOXO: doxorubicin; ZER: zerumbone; ZER-NLC: zerumbone-loaded nanostructured lipid carrier.

The LC_50_, TGI, and GI_50_ for ZER-NLC, ZER, and doxorubicin on the CMT cells are shown in Figure 8. Going by the 72-h LC_50_, TGI, and GI_50_ concentrations, doxorubicin was most lethal to CMT cells followed in order by ZER-NLC and ZER.

### 3.5. Eval**u**ation of Apoptosis Based on Cellular Morphology

**Figure 9:**
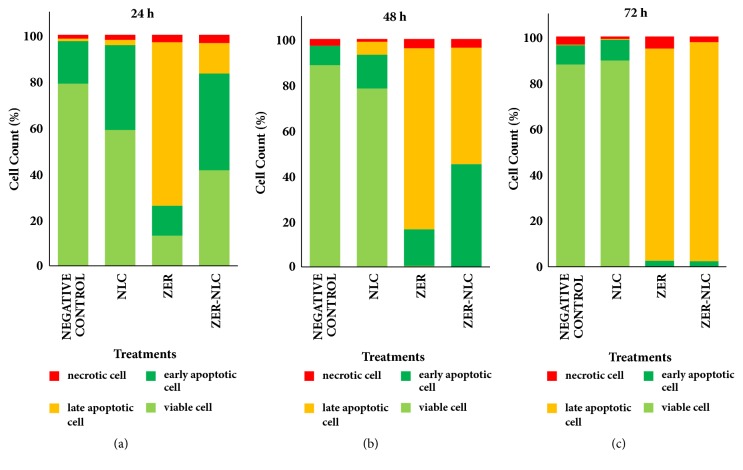
Viability and development of apoptosis and necrosis in canine mammary gland tumor (CMT) adenocarcinoma cells caused by ZER-NLC, ZER, and NLC treatments. ZER- and ZER-NLC-treated cells showed increase in late apoptotic cells with increasing period of treatment (*p*<0.0001). NLC: nanostructured lipid carrier; ZER: zerumbone; ZER-NLC: zerumbone-loaded nanostructured lipid carrier.

**Figure 10:**
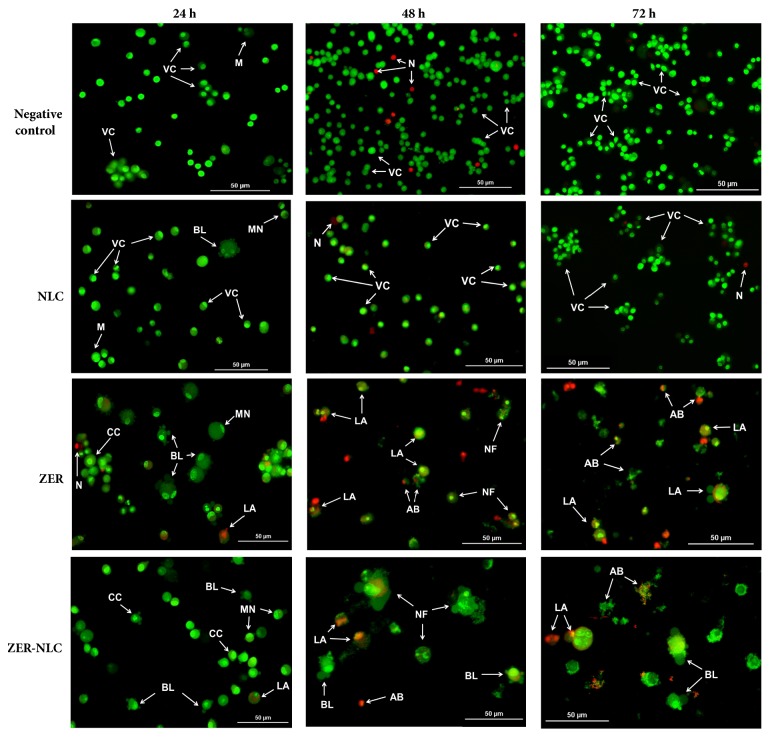
ZER-NLC-, ZER-, and NLC-treated canine mammary gland tumor (CMT) adenocarcinoma cells double-stained with acridine orange/propidium iodide (magnification: 20×). Negative control and NLC-treated cells showed greenish, intact normal cells with few reddish necrotic cells. Mitotic cells were seen in negative control and NLC-treated cells at 24 h. At 24 h, NLC-treated cells showed slight apoptotic cell morphology like cell membrane blebbing and marginated nucleus. At 24 h, ZER- and ZER-NLC-treated cells were in early apoptosis, evident by intercalated bright green staining, marginated nucleus, chromatin condensation, and cell membrane blebbing. At 48 and 72 h, ZER- and ZER-NLC-treated cells began to undergo late apoptosis, as shown by the apoptotic bodies and nuclear fragmentation. NLC: nanostructured lipid carrier; ZER: zerumbone; ZER-NLC: ZER-loaded NLC; VC: viable cells; LA: late apoptotic cell; N: necrotic cell; CC: chromatin condensation; MN: marginated nucleus; BL: cell membrane blebbing; M: mitosis; NF: nuclear fragmentation; AB: apoptotic bodies.

ZER-NLC and ZER caused time-dependent CMT cell death, with the effect becoming more severe with period of treatment (Figure 9). After 24 h, CMT cells treated with ZER-NLC and ZER showed condensed and marginated nuclear chromatin and membrane blebbing, indicating early apoptosis (Figure 10). By 48 h, these treated cells showed late apoptosis characterized by nuclear fragmentation (Figure 10). ZER produced greater rate of apoptosis than ZER-NLC on the CMT cells (Figure 9). Negative control cells were generally intact with occasional necrosis after 72 h of treatment. The NLC-treated CMT cells showed some degree of early apoptotic cell morphology at 24 h. This morphological change was not seen after 48 and 72 h of treatments.

### 3.6. Apoptotic Gene Expression

Linear standard curves with reasonable efficiency rate (E), coefficient of determination (R^2^), and slope were obtained for both Bax and Bcl-2 genes and reference RPS-19 and GAPDH genes (Figure S1). A single melting curve peak was observed for Bax, Bcl-2 genes, and the reference RPS-19 and GAPDH genes, respectively (Figure S2). This showed that there was only one PCR amplification product obtained for each PCR run, and the primers used were specific for the cDNA template. Standard and melting curves of real-time PCR are available in Supplementary Files S1-S2.

**Figure 11:**
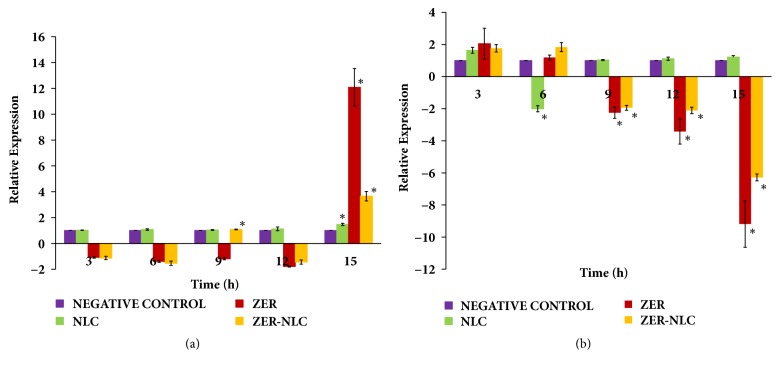
Gene expression, (a) Bax and (b) Bcl-2 genes, in canine mammary gland tumor (CMT) adenocarcinoma cells treated with ZER-NLC, ZER, and NLC. For each treatment, means with asterisk (*∗*) were significantly (*p*<0.05) different from their respective 3 h mean. ZER treatment: at 15 h, Bax gene expression increased significantly (p<0.0001) 12-fold while Bcl-2 gene expression decreased significantly (p<0.0001) 9-fold. ZER-NLC treatment: at 15 h, Bax gene expression increased significantly (p<0.0001) 4-fold while Bcl-2 gene expression decreased significantly (p<0.0001) 6-fold. NLC: nanostructured lipid carrier; ZER: zerumbone; ZER-NLC: zerumbone-loaded nanostructured lipid carrier.

ZER-NLC and ZER treatments significantly (*p*<0.0001) increased Bax and decreased Bcl-2 gene expressions in CMT cells (Figure 11). After 15 h of treatment, ZER produced a 12-fold increase and ZER-NLC a 4-fold increase in CMT cells' Bax gene expression. At the same time, ZER decreased Bcl-2 gene expression 9-fold and ZER-NLC 6-fold. The ZER treatment induced a much potent effect in Bax and Bcl-2 gene expressions as compared to ZER-NLC treatment. NLC had no appreciable effect on the CMT cell gene expression.

### 3.7. Caspase Protein Activation

**Figure 12:**
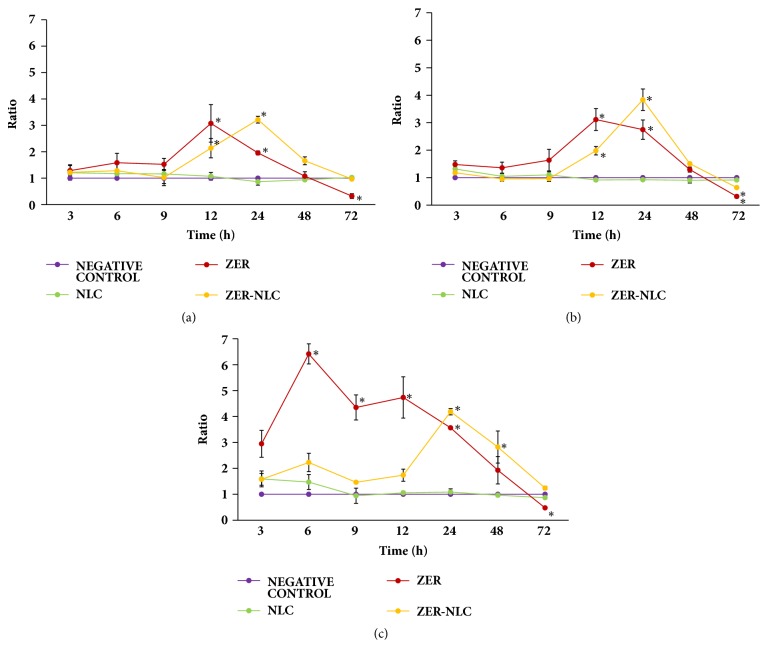
Caspase activities, (a) caspase-8, (b) caspase-9, and (c) caspase-3/7, in canine mammary gland tumor (CMT) adenocarcinoma cells treated with ZER-NLC, ZER, and NLC. Values are expressed as ratio to negative control. For each treatment, means with asterisk (*∗*) were significantly different (*p*<0.05) from their respective 3 h mean. ZER treatment: caspase-8 and -9 activities peaked significantly (*p*<0.0001) with 3-fold increase at 12 h while caspase-3/7 activity peaked significantly (*p*<0.0001) with 6-fold increase at 6 h. ZER-NLC treatment: caspase-8 and -9 activities peaked significantly (*p*<0.0001) with 3-fold increase at 24 h while caspase-3/7 activity peaked significantly (*p*<0.0001) with 4-fold increase at 24 h. NLC: nanostructured lipid carrier; ZER: zerumbone; ZER-NLC: zerumbone-loaded nanostructured lipid carrier.

ZER-NLC and ZER time-dependently increased caspase activities in CMT cells (Figure 12). The caspase-8 and -9 activities in CMT cells peaked significantly (*p*<0.0001) with 3-fold increase at 12 h of ZER treatment and similarly at 24 h of ZER-NLC treatment. Thereon, both caspase-8 and -9 activities decreased rapidly to almost no activity by 72 h (Figures 12(a) and 12(b)). Change in caspase-3/7 activity in ZER-treated CMT cells was even more rapid and potent, peaking with a significant 6-fold increase at 6 h (*p*<0.0001). However, ZER-NLC caused a more delayed effect on caspase-3/7 activity, peaking with significant 4-fold increase at 24 h (*p*<0.0001) (Figure 12(c)). The ZER treatment caused a much earlier effect on the caspases activation as compared to ZER-NLC treatment in the CMT cells. The NLC treatment did not induce appreciable caspase-8, -9, and -3/7 activities in the CMT cells.

## 4. Discussion

Although human medical research had progressed significantly over the last decades with breakthrough discoveries in innovative treatment regimens and chemotherapy drugs, the same is not true for veterinary medicine. In pet animal, for example, most cancer chemotherapeutics used are those developed for humans, largely from lack of specific studies on these animal diseases. In our study, the effects of ZER-NLC and ZER on a CMT cells were determined. The CMT cells used in our study were characterized via histopathology and PCR and shown to be of a primary CMT cell line. Histopathologically, the tumor was highly proliferative with malignant characteristics. The CMT cells were positive for the epithelial marker, cytokeratin-8; tumor markers, VEGF, HER-2, MMP-2, HIF-1*α*, and Bcl-2; and hormone receptors, PGR and EPOR, while negative they were for estrogen receptor, ER. Thus, the CMT cells used in this study were of mammary gland adenocarcinoma of epithelial origin, with malignant and invasive nature.

Treatment of CMT is by surgical excision with adjuvant chemotherapy and doxorubicin is usually included in chemotherapy for the advanced CMTs [3, 33]. However, doxorubicin causes adverse effects like cardiotoxicity, myelosuppression, gastrointestinal disorders, and hair follicle breakdown. Several phytochemicals were shown to have similar anticancer effects but without the unwanted side effects [34, 35]. Among these phytochemicals is ZER, a compound with promising anticancer and antiproliferative effects on numerous tumors including cervical cancer [14, 36], colon cancer [37, 38], liver cancer [39], pancreatic cancer [40], lung cancer [41], leukemia [8, 10, 22], mammary cancer [23, 24, 42], melanoma [43], and skin cancer [44]. However, the effect of ZER on canine tumors is still not known.

Zerumbone (ZER), a major lipophilic compound isolated from the essential oil of* Zingiber zerumbet *(L.) Smith rhizomes [9–12], is water-insoluble in its native form. Drugs with poor water solubility suffer from poor drug absorption which leads to inadequate and insufficient bioavailability [45]. This disadvantage renders ZER not suitable for* in vivo* parenteral application and, thus, limits its therapeutic application. To improve its bioavailability and efficacy, ZER was loaded into NLC and that rendered the compound water-soluble.

The ZER-NLC formulation was stable with long-term storage under 4°C, but not under 40°C storage. It is postulated that, at 40°C, the additional heat energy had caused the nanoparticles to grow and reduce in their surface charges (zeta potential) [46]. This eventually led to aggregation, flocculation, coagulation, or gelation of or a combination of these manifestations on the nanoparticles.

Lipid nanoparticle of approximately 50-100nm in size was previously reported to be large enough to exceed the glomerular capillary threshold of 10 nm [47] but small enough to escape elimination by immune cells, liver uptake, and clearance from circulation [48, 49]. Hence, freshly produced ZER-NLC, averaging 54.04 ± 0.19 nm in size, with slightly negative charges, was presumed to be able to access tumor tissues without hindrance following systemic administration [50]. These properties of ZER-NLC may allow for prolonged survival in blood circulation and improved bioavailability.

The efficaciousness of ZER-NLC as a cytotoxic compound was determined on the CMT cells. ZER-NLC, like ZER, significantly decreased proliferation of CMT cells in time- and concentration-dependent manners. The similarity in cellular response to ZER-NLC and ZER treatments showed that incorporation of ZER into NLC did not compromise the cytotoxic effect of ZER. However, overall ZER-NLC was more toxic than ZER to the CMT cells, suggesting the NLC may contribute to the cytotoxic effects of ZER-NLC [24]. This is also evident by the lower LC_50_, TGI, and GI_50_ of ZER-NLC than ZER on the cancer cells. It was postulated that the cytotoxic effect contributed by NLC is through its adherence to cell membranes, internalization, and degradation of cellular components [10].

It was observed that the CMT cell proliferation was greater with ZER than ZER-NLC treatment (Figure 7). It was postulated that cellular uptake of ZER was relatively slower than ZER-NLC. It is highly possible that the NLC of ZER-NLC had facilitated interaction between nanoparticle and cell membrane and allowed for more rapid internalization of the nanoparticle. By 72 h, presumably there was not much difference in amount of internalized ZER between cell treated with free ZER and ZER-NLC, thus the similarity in cytotoxic effects on the CMT cells. Furthermore, the NLC also has some degree of cytotoxic effect [18]. Thus, cytotoxic effect of ZER-NLC was due to the combined effect of NLC and ZER.

In an earlier study by our group, NLC, although insignificant, was shown to be slightly cytotoxic to the normal BALB/c 3T3 cells [18]. That study concluded that the cytotoxicity of hydrogenated palm oil (a component in NLC formulation) on the BALB/c 3T3 cells was found to be insignificant. Therefore, the inherent cytotoxicity of NLC was probably due to the nonionic surfactant, polysorbate 80. In general, surfactant has a detergenic effect disrupting the phospholipid bilayer of a cell, thus causing cell damage and reducing cell viability [51, 52]. Therefore, the polysorbate 80 component may render NLC slightly cytotoxic and non-specifically affecting both normal and cancer cells.

The CMT cells treated with NLC were still able to multiply until it reached confluency despite the cytotoxic characteristic it possesses, as evident in Figure 10 (at 72 h) in which NLC-treated CMT cells closely resembled negative control CMT cells in fluorescence micrograph imaging.

ZER-NLC and ZER induced time-dependent apoptosis on the CMT cells. Both treatments showed similar cell morphology of apoptosis. The population of treated CMT cells shifted from mainly early to late apoptosis with time of exposure (Figure 9). The induction of apoptosis by ZER-NLC was slower than ZER, suggesting that ZER was slowly released in a sustained manner from the ZER-loaded NLC nanoparticles. This is a desired characteristic of a drug carrier system because it allows for longer therapeutic effect with less dosing frequency.

Intrinsic and extrinsic pathways are the two main apoptotic pathways in mammalian cells. The intrinsic pathway is regulated by Bcl-2 family proteins and they can be either proapoptotic like Bax or antiapoptotic like Bcl-2 [53]. The Bcl-2/Bax protein in the mitochondria dictates the susceptibility of cancer cells to undergo apoptosis [54]. Bcl-2 overexpression blocks conformational change and translocation of Bax [55, 56] and it was proposed that Bcl-2 downregulation precedes Bax upregulation during the induction of apoptosis. This study supports the proposed theory as CMT cell apoptosis induced by ZER-NLC and ZER was via downregulation of Bcl-2 gene followed by upregulation of Bax gene expression.

Caspases, a family of cysteine proteases, are the central regulators of apoptosis. Caspase-3 and -7, with overlapping affinity towards key substrates responsible for DNA fragmentation and morphological apoptotic changes, are recognized as the hallmark enzymes of apoptosis [57]. Both ZER-NLC and ZER treatments induced significant (*p*<0.0001) increase in caspase-3/7 activity in CMT cells. But, the effect of ZER-NLC treatment on caspase-3/7 activity was more delayed than ZER (Figure 12). Similarly, the activities of caspase-8 and -9 peaked later with ZER-NLC than ZER treatment.

**Figure 13:**
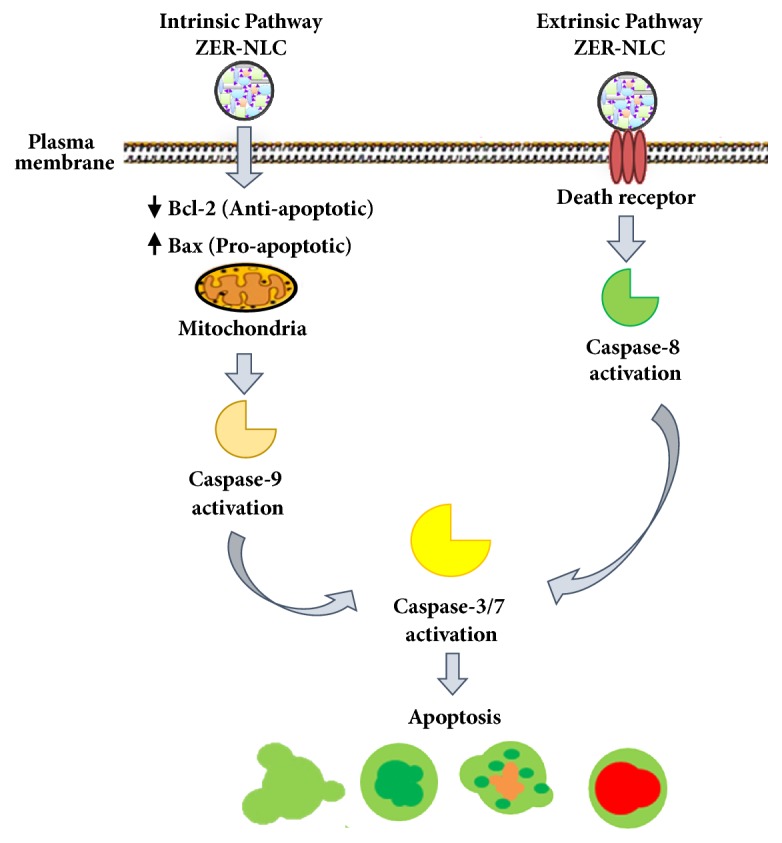
Hypothesis on the mechanism of effect of zerumbone-loaded nanostructured lipid carrier (ZER-NLC) on the canine mammary gland tumor (CMT) adenocarcinoma cells* in vitro*.

It is suggested that ZER-NLC activates caspase-8 by binding to the transmembrane death receptor on the CMT cell surface and directly activates caspase-3/7. Activated caspase-8 consequently induced mitochondria cytochrome-c released followed by activation of caspase-9, -3, and -7 [58].  Figure 13 shows hypothesis on the mechanism of effect of ZER-NLC on the CMT cells* in vitro*.

Although the gene expression and the activation of caspase suggested that ZER alone seems to be more effective compared to ZER-NLC in causing CMT cell apoptosis, the effect of ZER was earlier compared to that of ZER-NLC. This observation could be attributed to the sustained ZER release characteristic of ZER-NLC.

ZER is lipophilic and poorly water-soluble, which limits its therapeutic application. To improve ZER solubility, ZER was loaded into nanostructured lipid carrier (NLC) to produce ZER-loaded NLC (ZER-NLC). Our study showed that there was no significant difference between ZER and ZER-NLC treatment on the viability and development of apoptosis in CMT cells indicating that loading of ZER into NLC did not compromise the cytotoxic effect of ZER. In fact, the loading of ZER into NLC facilitates studies on its therapeutic application in diseases like cancers.

The most commonly used mammary gland tumor chemotherapeutics in dogs include doxorubicin, cyclophosphamide, and 5-fluorouracil, where doxorubicin is the most active agent for CMT patients with advanced disease [3, 33]. Unlike doxorubicin, ZER-NLC does not show appreciable* in vitro* toxicity towards normal human peripheral blood cells [15] or on the behavior, growth, serum biochemical profile, or organ histopathology of mice [59].

Even though* in vitro* ZER treatment in this study showed an earlier effect as compared to ZER-NLC treatment, ZER treatment will face hindrance for its clinical application due to its poor solubility and subsequent poor absorption and bioavailability [10]. Poorly water-soluble drugs often require higher doses in order to reach therapeutic plasma concentrations after oral administration [45]. Zerumbone (ZER), plagued with poor water solubility, will require a higher dose and more frequent dosing to reach therapeutic concentration in the body.

Most anticancer drugs cause serious adverse effects because of the sudden peak in concentration upon administration to a level higher than tolerable. At the same time, the concentration of free circulating drugs decreases rapidly. Thus, chemotherapy often requires frequent dosing to maintain therapeutic concentration in the body. The loading of ZER into NLC also serves to prolong circulation time of the compound, which improves uptake and accumulation in tumor tissues [60–62] and reduces requirement for frequent dosing. The sustained-released characteristic of ZER-NLC could prevent a sudden spike of the loaded drug in the plasma circulation, thereby lowering the risk of acute toxicity in the body [27]. ZER-NLC and ZER as a parenterally administered therapeutic were suggested to differ slightly in their modes of actions. ZER will immediately access target cells upon administration, while ZER-NLC must be internalized through either endocytosis, phagocytosis, or slow degradation of the nanocarrier to release ZER towards target tissues cells [10, 63]. This could account for the more rapid increase in Bax and decrease in Bcl-2 gene expression in ZER-treated compared to ZER-NLC-treated CMT cells. Circulating ZER-NLC degrades slowly through nanoparticle surface and bulk erosion, disintegration, diffusion, and desorption to release the loaded compound ZER from its nanocarrier ZER-NLC [10]. These features of ZER-NLC contribute to the sustained-released and extended therapeutic effects of ZER-NLC, making it an ideal anticancer drug carrier system.

## 5. Conclusion

The study showed that ZER-NLC and ZER were effective in inhibiting proliferation and inducing apoptosis on the CMT cells. The cytotoxic effects of ZER-NLC and ZER on the CMT cells were via inhibition of the antiapoptotic Bcl-2 and activation of proapoptotic Bax genes expressions and activation of caspases of the intrinsic and extrinsic apoptosis pathways. The effect of ZER-NLC as an anti-CMT compound was more gradual and sustained than ZER. The ZER-NLC with noncompromised ZER cytotoxic effect and added benefit of sustained drug release characteristic can potentially be developed as an innovative and safe delivery system for the treatment of CMT.

## Figures and Tables

**Table 1:** Primer sequences for the characterization of the canine mammary gland tumor (CMT) adenocarcinoma cell.

Genes	Primer Sequence (5' – 3')	Annealing Temperature (°C)
Cytokeratin-8 (CK-8)	Forward: AAGCAGCTTCTCCGCTCCTTCTA Reverse: CCAGCTTCAGTTTCTCCTAGCCCA	50

Hypoxanthine ribosyltransferase (HPRT)	Forward: TTA TCA GAC TGA AGA GCT ACT Reverse: TTA CCA GTG TCA ATT ATA TCT TCA ACA ATC	60

Estrogen Receptor (ER)	Forward: TCAGCTCTCGTCCTTCCTGCA Reverse: ACCAGACTCCATAATGGTAGCCTG	56

Progesterone Receptor (PGR)	Forward: AGGTGTACCAGCCGTACCTCAA Reverse: TTCGACCTCCAAGGACCATGCCAG	56

Vascular Endothelial Growth Factor Receptor (VEGF)	Forward: GCTCTCTTGGGTGCATTGGAGC Reverse: TAGGCCCTCATCATTACAGCAGC	58

Human epidermal growth factor receptor 2 (HER2)	Forward: ACCGGCACAGACATGAAGCTCC Reverse: AGCGGGTCTCCATTGTCCAGCA	50

Hypoxia-inducing Factor-1*α* (HIF-1*α*)	Forward: CGTTCCTTCGATCAGTTGTC Reverse: TCAGTGGTGGCAGTGGTAGT	56

B-cell lymphoma-2 (Bcl-2)	Forward: ATGTGTGTGGAGAGCGTCAACC Reverse: TGAGCAGAGTCTTCAGAGACAGCC	50

Matrix Metallopeptidase-2 (MMP-2)	Forward: CCACGTGACAAGCCCATGGGGCCCC Reverse: GCAGCCTAGCCAGTCGGATTTGATG	60

Erythropoietin Receptor (EPOR)	Forward: CCTGACGCTCTCCCTCATCC Reverse: GCCTTCAAACTCGCTCTCTGG	65

**Table 2:** LC_50_, TGI, and GI_50_ values for canine mammary gland tumor (CMT) adenocarcinoma cells treated with ZER-NLC, ZER, DOXO.

Concentration (*μ*M)		Treatments		
	Time (h)	ZER-NLC	ZER	DOXO
LC_50_	24	84.70 ± 8.68	128.68 ± 5.47	95.99 ± 10.54
	48	68.61 ± 10.10	84.86 ± 14.30	19.62 ± 5.83
	72	84.54 ± 10.44	94.63 ± 10.80	5.89 ± 0.05

TGI	24	20.47 ± 10.52	46.22 ± 10.12	24.91 ± 10.88
	48	23.52 ± 6.87	34.39 ± 5.87	4.51 ± 0.08
	72	27.29 ± 4.87	32.28 ± 1.29	3.93 ± 0.03

GI_50_	24	4.12 ± 0.97	18.49 ± 3.63	4.39 ± 0.77
	48	4.32 ± 0.71	11.44 ± 2.48	2.25 ± 0.04
	72	8.63 ± 1.16	11.36 ± 0.42	1.96 ± 0.02

Data are mean ± SD (n=3), analyzed using one-way ANOVA and* post hoc *Tukeytest.

DOXO: doxorubicin; GI_50_: growth inhibition concentration 50; LC50: lethal concentration 50; TGI: total growth inhibition concentration; ZER: zerumbone; ZER-NLC: zerumbone-loaded nanostructured lipid carrier.

**Table 3:** Primer sequences for PCR analysis of apoptotic-related and reference genes.

Gene	Forward and reverse primers (5' – 3')	Annealing Temperature (°C)	Product length (bp)	Accession number
Bcl-2	Forward: TGG ATG ACT GAG TAC CTG AA Reverse: GGC CTACTG ACT TCA CTT AT	56.00	206.00	AB116145

Bax	Forward: GGT TGT TGC CCT CCT CTA CT Reverse: GTA AGC ACT CCA GCC ACA AA	60.00	219.00	AB080230

RPS-19	Forward: CCT TCC TCA AAA AGT CTG GG Reverse: GTT CTC ATC GTA GGG AGC AAG	61.00	95.00	XM_533657

GAPDH	Forward: TGT CCC CAC CCC CAA TGT ATC Reverse: CTC CGA TGC CTG CTT CAC TAC CTT	58.00	100.00	NM_001003142

**Table 4:** Molecular markers for murine (3T3) fibroblast and canine mammary gland tumor (CMT) adenocarcinoma cells.

Genes	Gene Expression	
	3T3 Murine fibroblast	Canine mammary gland adenocarcinoma cell
CK-8	-	+
HPRT	+	+
ER	-	-
PGR	-	+
VEGF	-	+
HER-2	-	+
HIF-1*α*	-	+
Bcl-2	-	+
MMP-2	-	+
EPOR	-	+

-: negative; +: positive; CK-8: cytokeratin-8; HPRT: hypoxanthine ribosyltransferase; ER: estrogen receptor; PGR: progesterone receptor; VEGF: vascular endothelial growth factor; HER-2: human epidermal growth factor receptor-2; HIF-1*α*: hypoxia-inducing factor-1*α*; Bcl-2: B-cell lymphoma-2; MMP-2: matrix metalloproteinases-2; EPOR: erythropoietin receptor.

## Data Availability

The data used to support the findings of this study are available from the corresponding author upon request.

## Abbreviations

AOPI: Acridine orange and propidium iodide
Bcl-2: B-cell leukemia/lymphoma-2
bp: Base pair
CK-8: Cytokeratin-8
CMT: Canine mammary gland tumor
DMSO: Dimethyl sulfoxide
DOXO: Doxorubicin
EPOR: Erythropoietin receptor
ER: Estrogen receptor
GI_50_: Growth inhibition concentration 50
H&E: Hematoxylin and eosin
HER-2: Human epidermal growth factor receptor-2
HIF-1*α*: Hypoxia-inducing factor-1*α*
HPRT: Hypoxanthine ribosyltransferase
LC_50_: Lethal concentration 50
MMP-2: Matrix metalloproteinases-2
NLC: Nanostructure lipid carrier
PCR: Polymerase chain reaction
PDI: Polydispersity index
PGR: Progesterone receptor
SLN: Solid lipid nanoparticles
TGI: Total growth inhibition concentration
VEGF: Vascular endothelial growth factor
ZER: Zerumbone
ZER-NLC: Zerumbone-loaded nanostructure lipid carrier
ZP: Zeta potential.

## Supplementary Materials

Supplementary Materials Figure S1: quantitative reverse transcription real-time PCR (qRT-PCR) standard curves for canine mammary gland tumor (CMT) adenocarcinoma cells for (a) Bax, (b) Bcl-2, (c) reference RPS-19, and (d) GAPDH genes. Figure S2: quantitative reverse transcription real-time PCR (qRT-PCR) melting curves for canine mammary gland tumor (CMT) adenocarcinoma cells for (a) Bax, (b) Bcl-2, (c) reference RPS-19, and (d) GAPDH genes.
