# Supplementary material for: Zerumbone-Loaded Nanostructured Lipid Carrier Induces Apoptosis of Canine Mammary Adenocarcinoma Cells
Source: Biomed Res Int. 2018 Oct 15;2018:8691569. doi: 10.1155/2018/8691569 (PMC6205321; doi:10.1155/2018/8691569)
Supplement: Supplementary Materials — Figure S1: quantitative reverse transcription real-time PCR (qRT-PCR) standard curves for canine mammary gland tumor (CMT) adenocarcinoma cells for (a) Bax, (b) Bcl-2, (c) reference RPS-19, and (d) GAPDH genes. Figure S2: quantitative reverse transcription real-time PCR (qRT-PCR) melting curves for canine mammary gland tumor (CMT) adenocarcinoma cells for (a) Bax, (b) Bcl-2, (c) reference RPS-19, and (d) GAPDH genes. [file 8691569.f1.docx]

Manuscript ID: 8691569 version 3

Manuscript Title: Zerumbone-loaded Nanostructured Lipid Carrier Induces Apoptosis of Canine Mammary Adenocarcinoma Cells.

# SUPPLEMENTARY FILE:


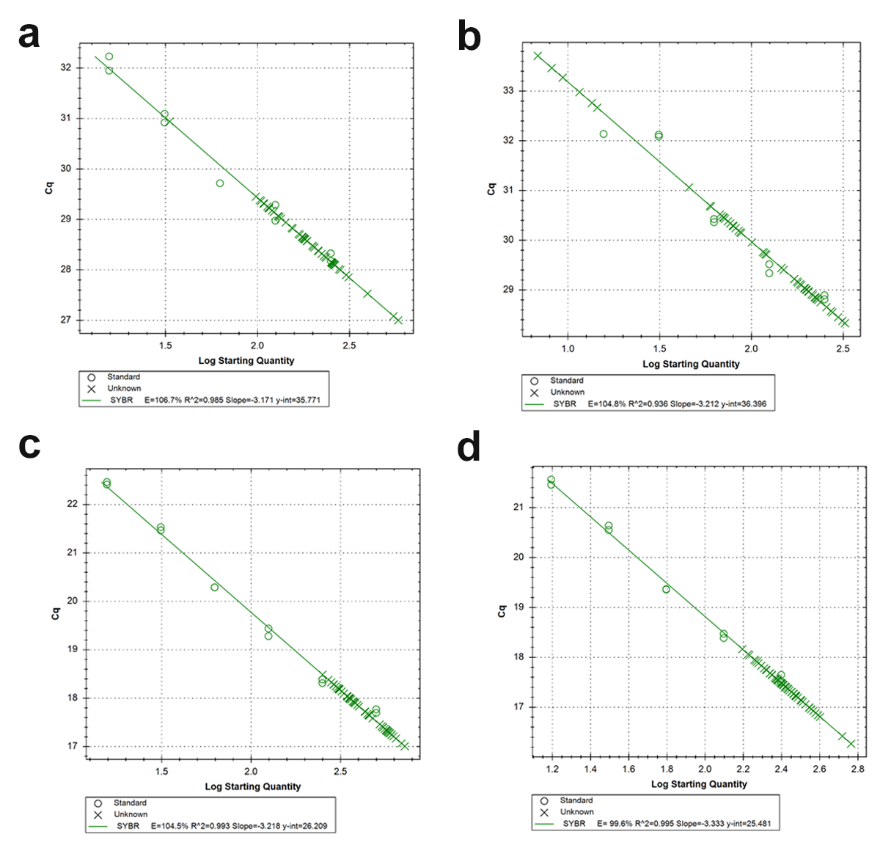


### Figure S1: Quantitative reverse transcription real-time PCR (qRT-PCR) standard curves for canine mammary adenocarcinoma cells for (a) Bax, (b) Bcl-2, (c) reference RPS-19, and (d) GAPDH genes.


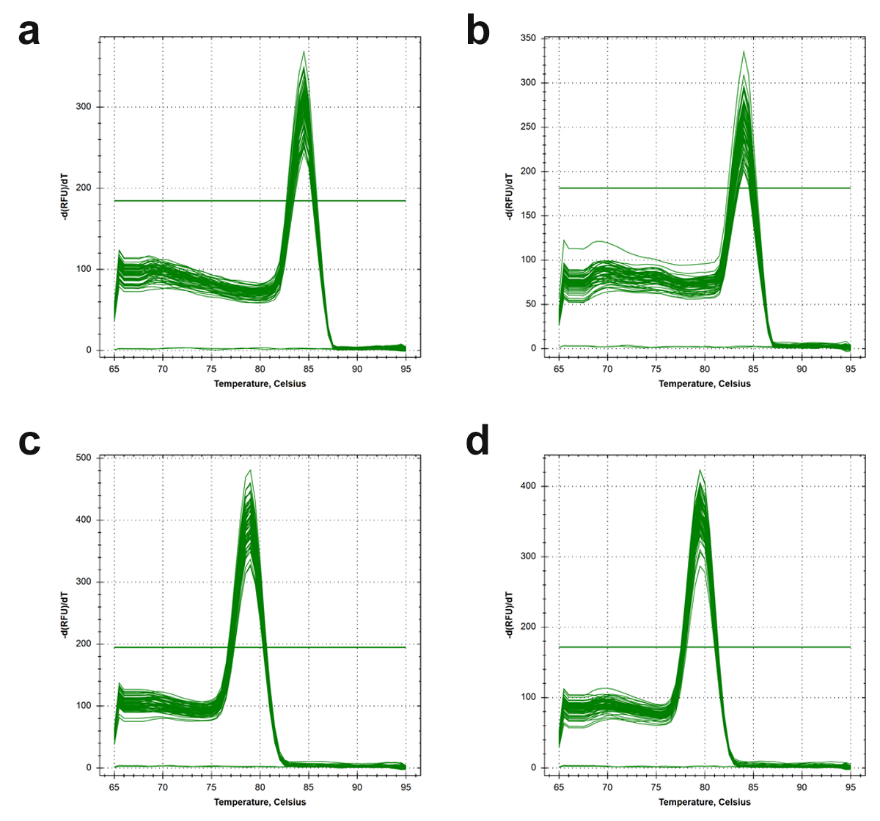


### Figure S2: Quantitative reverse transcription real-time PCR (qRT-PCR) melting curves for canine mammary adenocarcinoma cells for (a) Bax, (b) Bcl-2, (c) reference RPS-19 and (d) GAPDH genes.
